# High-Value and Environmentally Friendly Recycling Method for Coal-Based Solid Waste Based on Polyurethane Composite Materials

**DOI:** 10.3390/polym16142044

**Published:** 2024-07-17

**Authors:** Xu Li, Yang Liu, Mingyi Li, Sitong Zhang, Lan Jia, Fengbo Zhu, Wenwen Yu

**Affiliations:** 1 College of Materials Science & Engineering, Taiyuan University of Technology, Taiyuan 030024, China; lixu0185@link.tyut.edu.cn (X.L.); liuyang0280@link.tyut.edu.cn (Y.L.); 13703573387@163.com (M.L.); 2023310075@link.tyut.edu.cn (S.Z.); zhufengbo@tyut.edu.cn (F.Z.); yuwenwen@tyut.edu.cn (W.Y.); 2 Shanxi-Zheda Institute of Advanced Materials and Chemical Engineering, Taiyuan 030000, China

**Keywords:** composite materials, polyurethane, coal gangue, fly ash

## Abstract

This study aims to provide a high-value and environmentally friendly method for the application of coal-based solid waste. Modified fly ash/polyurethane (MFA/PU) and modified coal gangue powder/polyurethane (MCG/PU) composites were prepared by adding different contents of MFA and MCG (10%, 20%, 30%, 40%). At the filler content of 30%, the compressive strengths of MFA/PU and MCG/PU are 84.1 MPa and 46.3 MPa, respectively, likely due to an improvement in interface compatibility, as indicated by scanning electron microscopy (SEM). The MFA/PU and MCG/PU composites present their highest limiting oxygen index (LOI) values of 29% and 23.5%, respectively, when their filler content is 30%. MFA has advantages in improving the LOIs of composites. Cone calorimetry (CCT) and SEM demonstrate that the two composites exhibit similar condensed-phase flame-retardant behaviors during combustion, which releases CO_2_ in advance and accelerates the formation of a dense barrier layer. Compared with the MFA/PU composites, the MCG/PU composites could produce a more stable and dense barrier structure. Water quality tests show that heavy metals do not leak from FA and CG embedded in PU. This work provided a new strategy for the safe and high-value recycling of coal-based solid waste.

## 1. Introduction

China, as the world’s largest coal producer and consumer, faces significant challenges in dealing with the accumulation of coal-based solid wastes such as coal gangue (CG) and fly ash (FA). These wastes not only occupy valuable land resources but also cause severe environmental problems [1,2]. In recent years, there has been a growing focus on finding sustainable solutions for utilizing these solid wastes due to increased awareness of the need for sustainable development and the rising demand to address their accumulation issues [3,4]. While CG has a higher yield compared to FA, its recovery and reuse are more challenging due to cost implications associated with crushing. Currently, CG is primarily used for filling mining areas, producing building materials, and generating power [5,6,7], limiting its application in high-value products [8,9].

Polyurethane (PU) materials have excellent mechanical properties, chemical and wear resistance, and processability. Their products are widely used in textiles, construction, aviation, medicine, electronics, and other fields [10,11,12,13]. However, the flammability and inadequate flame retardancy of PU materials also restrict their usage in various domains [14]. Adding flame retardants is a common method to improve the flame retardancy of PU materials [15]. Some commonly used flame retardants produce a large amount of smoke or toxic gases during combustion, causing harm to human health and the environment [16]. Inorganic fillers, such as silicate [17,18], hydroxide [19], and FA [20,21], have the characteristics of nontoxicity, low price, and good flame retardancy. These characteristics can considerably improve the flame retardancy of PU materials and reduce costs. Therefore, inorganic fillers are an effective way to improve the fire safety of PU materials.

Previous studies have utilized FA as a flame-retardant filler in polymer materials, demonstrating its ability to enhance the thermal stability, hydrophobicity, and flame retardancy of PU materials [22]. Furthermore, research has shown that FA and certain mineral fillers can collaborate with flame retardants to facilitate carbon layer formation [23,24,25]. In terms of composition, CG contains a small amount of carbon (typically < 20%) and trace minerals. Its other components are similar to those found in FA and primarily consist of SiO_2_, Al_2_O_3_, and other oxides, which also hold potential as fillers. As fillers, FA and CG generally need to be modified to improve their compatibility with the matrix.

The main objective of this study is to develop an eco-friendly and high-value approach for utilizing coal-based solid waste in the production of PU matrix composites. Modified FA/PU (MFA/PU) and modified CG/PU (MCG/PU) composites with varying filler contents were prepared by incorporating a silane coupling agent, MFA, and MCG powder into a PU matrix. The impact of filler modification on the mechanical properties of the composites was investigated through changes in the compressive strength and cross-section morphology. Furthermore, the thermal stability and combustion properties of the composites were analyzed using thermogravimetric (TG) analysis, limiting oxygen index (LOI), and CCT, while scanning electron microscopy (SEM) was employed to observe the morphology of residual composites after combustion. The flame-retardant mechanism of both composites was analyzed and summarized. Finally, heavy metal leakage from the composites was evaluated by examining their influence on water quality.

## 2. Materials and Methods

### 2.1. Materials

Polyether polyol PPG-2000 (hydroxyl value 51–62 mg KOH/g, industrial grade) was obtained from Shanghai Titan Technology Co. (Shanghai, China). Polyether polyol RT-305 (hydroxyl value 320–340 mg KOH/g, industrial grade) was purchased from Nantong-Ruitai Chemical Co. (Nantong, China). FA (300 mesh, industrial grade) was provided by Henan Platinum Casting Materials Co., Ltd. (Zhengzhou, China), CG powder (300 mesh, industrial grade) was acquired from Henan Lanke Water Purification Material Co. (Zhengzhou, China). 3-Glycidyl ether oxypropyl-trimethoxysilane (KH-560, purity 97%) was procured from Shanghai McLean Biochemical Technology Co., Ltd. (Shanghai, China). Anhydrous ethanol (AR) was bought from Shanghai McLean Biochemical Technology Co. (Shanghai, China). Dibutyltin dilaurate (DBTDL, purity 95%) was purchased from Shanghai Aladdin Biochemical Technology Co. (Shanghai, China). Polymethylene polyphenyl polyisocyanate (PAPI, industrial grade) was purchased from Shandong Yantai Wanhua Co. (Yantai, China).

### 2.2. Surface Modification of FA and CG Powder

Aqueous ethanol solution (ethanol to water: 9:1 by volume) was prepared, then 80 g CG or FA powder was added to 200 mL ethanol aqueous solution. Subsequently, the solution had 2.5 wt.% KH-560 added and was stirred at 500 rpm. The modification time of CG powder or FA was 60 min. MFA and MCG precipitates were obtained through centrifugation. Finally, after vacuum drying at 80 °C for 24 h, MCG powder and MFA powder were obtained by grinding.

### 2.3. Preparation of PU Composites

**Figure 1:**
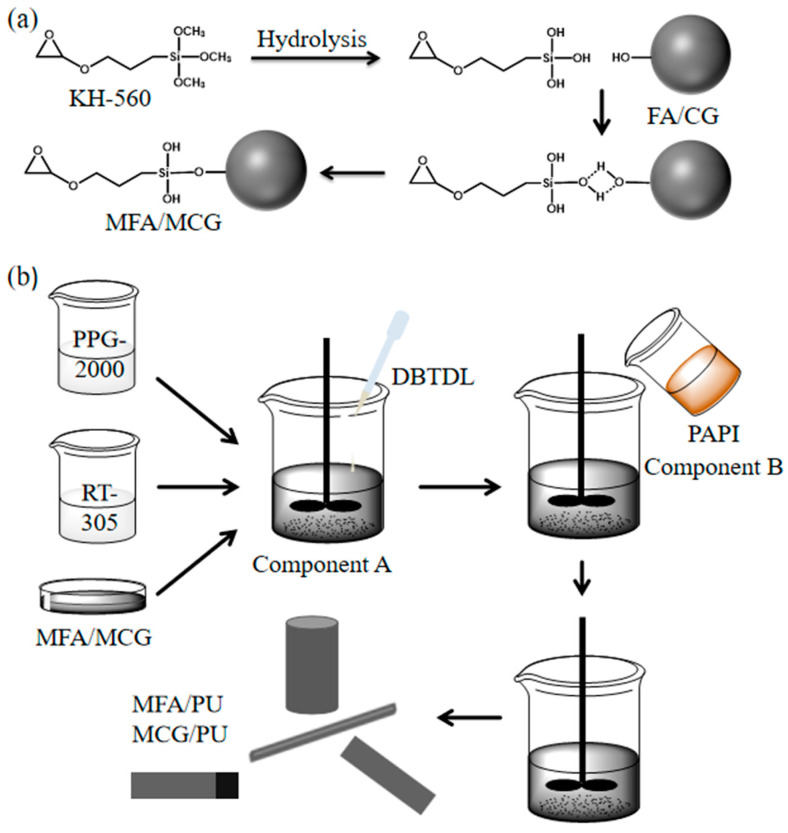
The scheme of (**a**) surface modification of FA and CG powder and (**b**) preparation of MFA/PU and MCG/PU composite materials.

The preparation of MFA/PU and MCG/PU composites is shown in Figure 1. MFA and MCG were dried in a vacuum oven at 110 °C for 2 h and sealed for preservation. PPG-2000, dried and sealed MFA and MCG, and the catalyst DBTDL were added into a cup filled with RT-305 and mixed uniformly at 500 rpm. This mixture was recorded as component A. PAPI was weighed and recorded as component B. Polyurethane is a kind of block polymer composed of alternating hard and soft segments. During the synthesis of PU composites, PPG-2000 and RT-305 are polyether polyols that form the soft segment in polyurethanes, while PAPI is the hard segment in polyurethanes. After the samples were cured, MFA/PU and MCG/PU composites were obtained by mixing the A and B components at 500 rpm. The specific formulations of MFA/PU and MCG/PU composites with different content fillers are shown in Table 1.

PU, FA/PU, and CG/PU composites were prepared through the above process without adding fillers (MFA or MCG) and unmodified fillers (FA and CG) for comparative analysis. The preparation processes of PU, FA/PU, and CG/PU composites were similar to those shown in Figure 1.

### 2.4. Testing and Characterization

FA and CG powder samples were analyzed by using X-ray diffractometer (XRD, MAX-2600, Rigaku D, Tokyo, Japan) to study the differences in phase composition between the two fillers.

FA and CG powder samples were analyzed by using X-ray fluorescence (XRF, Panalytic Axios, Amsterdam, Netherlands) to study their chemical composition after calcination and to compare the differences in their compositions.

In accordance with the GB/T 2567-2008 standard [26], cylindrical specimens with a diameter of 50 mm and height of 100 mm were prepared, and the compressive strength of the two composites was tested by using a universal testing machine (Z25003051242, Paxen, Germany) at a compression speed of 5 mm/min.

The thermal stability of the PU, MFA/PU, and MCG/PU composites was measured by using a TG analyzer (NETZSCH TG-209, Bavaria, Germany) at 30–900 °C and a heating rate of 10 °C/min under a N_2_ protective atmosphere.

In accordance with the GB/T 2406.2-2009 standard [27], 15 mm × 10 mm × 4 mm samples were prepared, and the LOIs of the PU, MFA/PU, and MCG/PU composites were measured by an LOI analyzer (PX-01-005, Phoenix, Suzhou, China).

A sample with dimensions of 100 mm × 100 mm × 8 mm was prepared. A cone calorimeter (FTT-0242, FTT, East Grinstead, UK) was used for CCT under the conditions of 20.9% oxygen concentration and 35 kW/m^2^ heat flux, respectively.

SEM (Gemini SEM 360, Carl Zeiss, Oberkochen, Germany) was used to investigate the particle morphology of FA and CG fillers, cross-section morphology of the composites before and after modification, and change in carbon residue after CCT.

The water quality test was performed as follows: a cylindrical specimen with a diameter of 50 mm and height of 100 mm was soaked for 24 h in a container containing 20 L of domestic water at 23 ± 2 °C. The water used to soak the sample was collected to determine the influence of the composites on water quality in accordance with the GB/T 5750.1-2023 standard [28]. Test results were evaluated in accordance with the requirements of the GB 5749-2022 standard for domestic water [29].

## 3. Results and Discussion

### 3.1. Composition and Morphological Analyses of FA and CG

**Figure 2:**
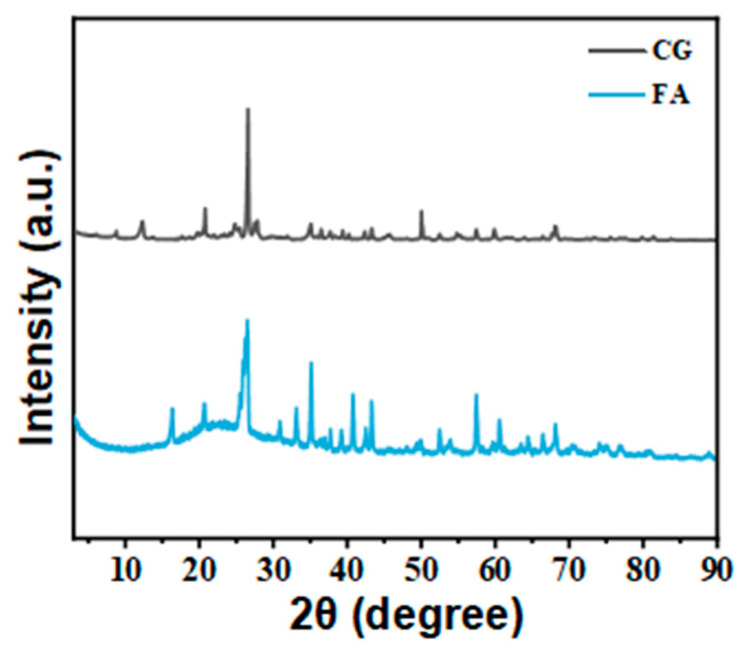
XRD profiles of FA and CG.

The phase composition of FA and CG was analyzed using XRD, as shown in Figure 2. It can be found that there are significant differences in the diffraction patterns of the two coal-based solid waste powders. Further analysis was conducted on the specific phase composition of FA and CG, and the results are shown in Table 2.

Due to CG being a byproduct of raw coal mining, its phase composition is relatively complex, and is mainly composed of crystal structures such as quartz, kaolinite, illite, and plagioclase, with the exception of noncrystalline materials. Low-strength clay minerals such as kaolin and illite can influence the reinforcement effect of CG fillers; as a byproduct of coal or coal gangue co-firing, it is mainly composed of a mullite phase, quartz, and amorphous substances, with significant differences in phase composition compared to CG.

XRF was used to analyze the chemical compositions of FA and calcined CG, and the results are shown in Table 3. FA and calcined CG are mainly composed of SiO_2_, Al_2_O_3_, Fe_2_O_3_, and CaO and contain a small amount of SO_3_ (as CaSO_4_) and other metal oxides. In this study, the sum of the contents of SiO_2_, Al_2_O_3,_ and Fe_2_O_3_ in FA and CG are 90.41 and 81.58%, respectively. Thermogravimetric testing showed that the loss of ignition of the CG used in this study is approximately 9.7%. According to the literature, the content of SiO_2_, Al_2_O_3,_ and Fe_2_O_3_ oxides in CG exceeds 80% [30,31]. This characteristic can improve the mechanical properties and fire resistance of polymers. Although the compositions of the two materials share similarities, their differences also lead to differences in the mechanical strength and flame retardancy of their composites.

**Figure 3:**
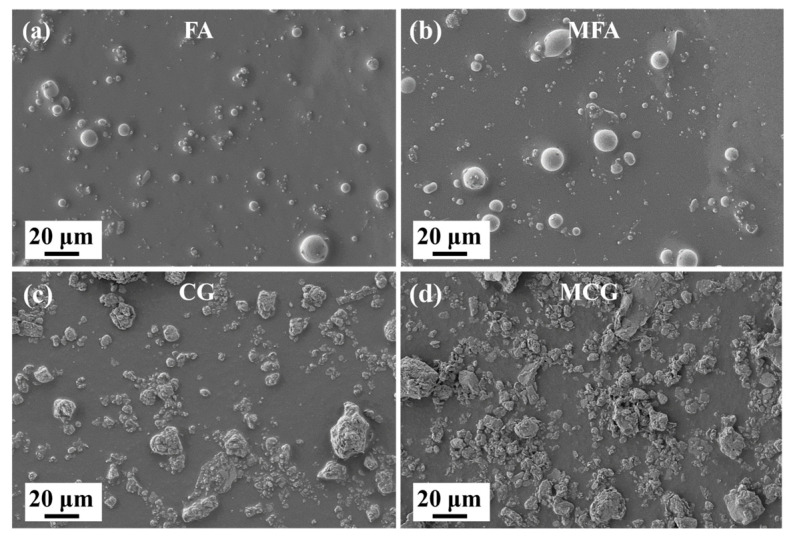
SEM images of (**a**) FA, (**b**) CG, (**c**) MFA, and (**d**) MCG, with a magnification of 500×.

SEM was used to observe the particle morphologies of FA and CG before and after modification. Figure 3 shows that the particle morphologies of the two fillers are different. FA is composed of spherical particles and a few massive particles, whereas CG powder is composed of some irregular and rough amorphous particles. The average sizes of FA and CG were measured to be 5 μm and 7 μm, respectively. FA and CG have not undergone significant morphological changes before and after modification.

### 3.2. Compressive Strength of Composites

**Figure 4:**
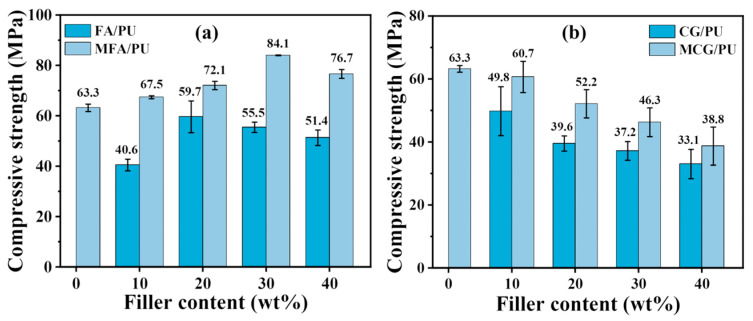
Compressive strengths of composites before and after filler modification depending on filler content: (**a**) FA/PU and MFA/PU, (**b**) CG/PU and MCG/PU.

The compressive strength of the FA/PU and CG/PU composites before, and MFA/PU and MCG/PU after the modification of the two fillers was tested to explore the influence of FA and CG on the composites. Figure 4 illustrates that the compressive strengths of the two composites show different trends with the increase in the filler content. With the increase in the FA mass fraction, the compressive strength of the composites first increases and then decreases. With the increase in the CG mass fraction, the compressive strength of the composites gradually declines. After modification, the compressive strength of the MFA/PU and MCG/PU composites has improved relative to that of the FA/PU and CG/PU composites.

At the filler content of 30%, the compressive strengths of the MFA/PU and MCG/PU composites are 84.1 and 46.3 MPa, respectively. Compared with the compressive strength of pure PU (63.3 MPa), that of the MFA/PU composites has increased by 33%, while that of the MCG/PU composites has decreased by 27%. Both composites still exhibit good mechanical strength. The high compressive strength of the MFA/PU and MCG/PU composites indicates that the modified fillers can be dispersed well in the PU matrix and the interface among MFA, MCG, and the PU matrix is tightly bonded. Compared with the unmodified fillers, the compatibility between the modified fillers and the matrix is improved, thus enhancing the compressive strength of the two composites. Stress concentration appears in the composites under the action of external forces. This phenomenon leads to a decrease in the compressive strength. The transfer of the peak compressive strength of the MFA/PU composite to a high filler content also indicates that the modified fillers could improve filler dispersion in the PU matrix. In addition, organic carbon and clay minerals in the CG filler affect its reinforcement effect [3,32]. Under the action of external forces, CG becomes a stress concentration point when present at high contents, resulting in a low compressive strength.

However, composites incorporating CG/MCG as a filler demonstrated a superior fracture toughness compared to those utilizing FA/MFA as a filler. As the filler content increased from 10% to 40%, the elongation at break of the FA/CG and MFA/CG composites decreased from approximately 60% to around 45%, whereas the reduction in the elongation at break for the CG/PU and MCG/PU composites was relatively smaller, ranging from about 65% to about 55%. The corresponding results are illustrated in Figures S1 and S2. This could be attributed to the relatively lower strength of cg compared to FA, while exhibiting superior energy absorption characteristics in comparison with FA. Nevertheless, the compressive strength of the MCG/PU composites with 30% MCG is sufficient to meet most of the needs of the reinforcements, including mine, slope, and road reinforcements.

### 3.3. Analysis of the Cross-Section Morphologies of Composites

**Figure 5:**
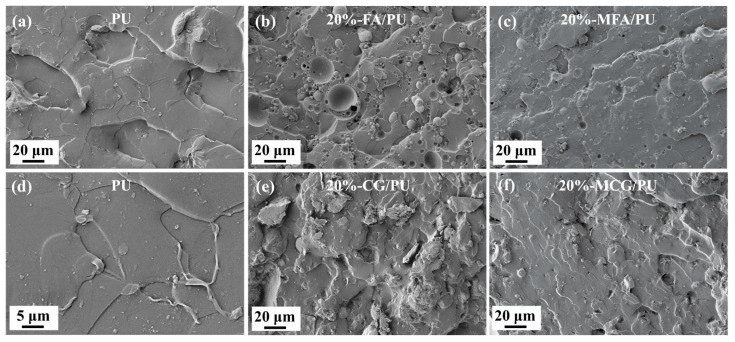
Cross-section morphologies of (**a**) pure PU (500×), (**b**) FA/PU composite with 20% FA (500×), (**c**) MFA/PU composite with 20% MFA (500×), (**d**) pure PU (2000×), (**e**) CG/PU composite with 20% CG (500×), and (**f**) MCG/PU composite with 20% MCG (500×).

The cross-section morphologies of the PU, 20%-FA/PU, and 20%-CG/PU composites before and after modification with the two fillers were compared by using SEM. Figure 5 shows that the surface of the PU material is smooth and flat with uneven fracture morphology in some areas. The surfaces of the 20%-FA/PU and 20%-CG/PU composites are rough, exhibiting irregular protrusions, uneven dispersion in some areas, and aggregations when CG and FA were added to the composites before modification. The surface morphology of the 20% -MFA/PU and 20%-MCG/PU composites modified with two kinds of fillers is different from that of the 20%-FA/PU and 20%-CG/PU composites. The composites not only have the smooth cross-section morphology of PU materials, but they also have relatively uniform protrusions and good filler dispersion. This phenomenon shows that MFA and MCG are dispersed well in the two composites, coated well by the PU matrix, and bound closely to the interface. This effect accounts for the improved compressive strength of the modified composites.

### 3.4. Limiting Oxygen Index

The LOI values of PU and the composites are listed in Table 4. Pure PU is a flammable material, and its LOI is only 19%. The LOI values of the MFA/PU and MCG/PU composites increase first and then decrease with the increase in the MFA and MCG filler contents. When the MFA mass fraction exceeds 10%, the composite MFA/PU changes from a flammable material into a slow-burning material; when the MFA mass fraction is 30%, the LOI values reach the maximum values of 29% and the flame retardancy of the MFA/PU composite reaches the flame-retardant level. For the MCG/PU composites, when the MCG mass fraction exceeds 20%, the composite will change from a flammable material to a slow-burning material. The maximum oxygen index value is 23.5% with 30% MCG, and the composite is still a slow-burning material.

The increase in the LOI of the two composites indicates that the addition of modified fillers can substantially improve the flame retardancy of PU. The LOI of the MFA/PU composites is higher than that of the MCG/PU composites, and the flame retardancy of MCG fillers greatly affects the flame retardancy of the composites. Although MCG and MFA have similar compositions, the organic components in uncalcined MCG spontaneously ignite at high temperatures and in the presence of sufficient O_2_ and promote the combustion of the MCG/PU composites. This phenomenon is the main reason why the oxygen index of the MCG/PU composites increases negligibly. However, FA calcined at a high temperature coats the surfaces of combustible materials to form a barrier layer when the MFA/PU composites burn. This layer absorbs heat and prevents O_2_ from coming into contact with combustible materials, thus greatly improving the flame retardancy of the composites.

### 3.5. Thermal Stability

**Figure 6:**
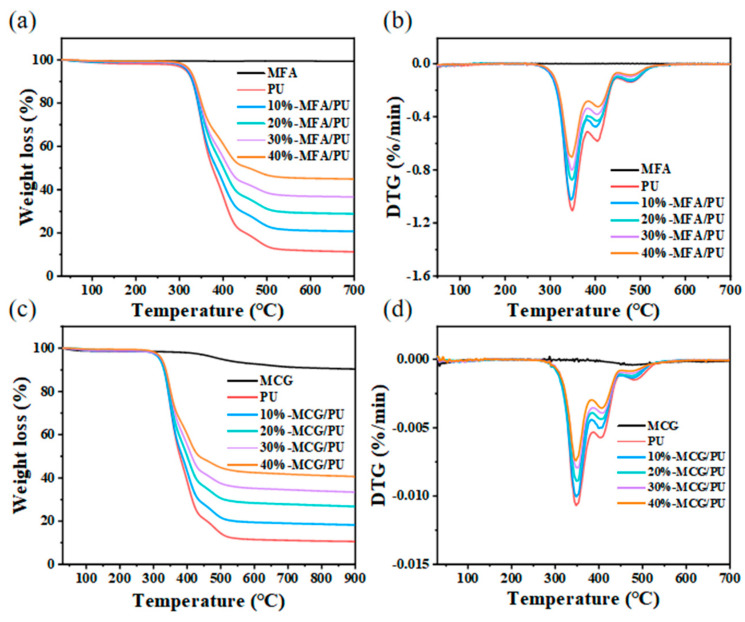
TG (**a**) and DTG (**b**) curves of MFA/PU composite materials; TG (**c**) and DTG (**d**) curves of MCG/PU composite materials.

The thermogravimetric analysis (TG) and derivative thermogravimetry (DTG) curves of the modified fillers (MFU and MCG), PU, and composites (MFA/PU and MCG/PU) with different filler amounts (10%, 20%, 30%, 40%) were acquired under N_2_ atmosphere to further understand the influence of filler addition on the thermal stability of the composites. The results are shown in Figure 6. The pyrolysis behaviors of MFA and MCG fillers are different. MFA does not degrade. The pyrolysis of organic carbon in MCG occurs at approximately 400 °C [30], and the content of organic carbon in MCG accounts for approximately 10% of its mass, which is also the reason for the low limiting oxygen index of the MCG/PU composite materials. The thermal degradation of PU includes three stages: the first stage occurs between 300 °C and 380 °C and is mainly the decomposition of carbamate in PU. The second stage occurs at approximately 380–450 °C and corresponds to the destruction of the polyol long chain. The third stage is the further destruction of the polyol short chain at approximately 450–500 °C.

Figure 6b,d show that the thermal degradation of the MFA/PU and MCG/PU composites still has three stages. The thermal degradation of the MFA/PU composites is similar to that of PU, and the addition of MFA barely affects the decomposition temperature of PU. The third pyrolysis peak of the MCG/PU composites has shifted slightly to the left. This behavior may be related to the pyrolysis of organic components in MCG. With the increase in filler content, the pyrolysis rates of the two composites decrease and the amount of pyrolysis residues increase. This effect may be related to the increase in oxides, such as SiO_2_ and Al_2_O_3_, in the composites [33,34]. These oxides can absorb heat and form a barrier layer during the pyrolysis of the composites. This residual barrier layer hinders the heat transfer and thermal decomposition of PU, thus increasing the residual amount of composites after pyrolysis.

### 3.6. Cone Calorimetric Test

**Figure 7:**
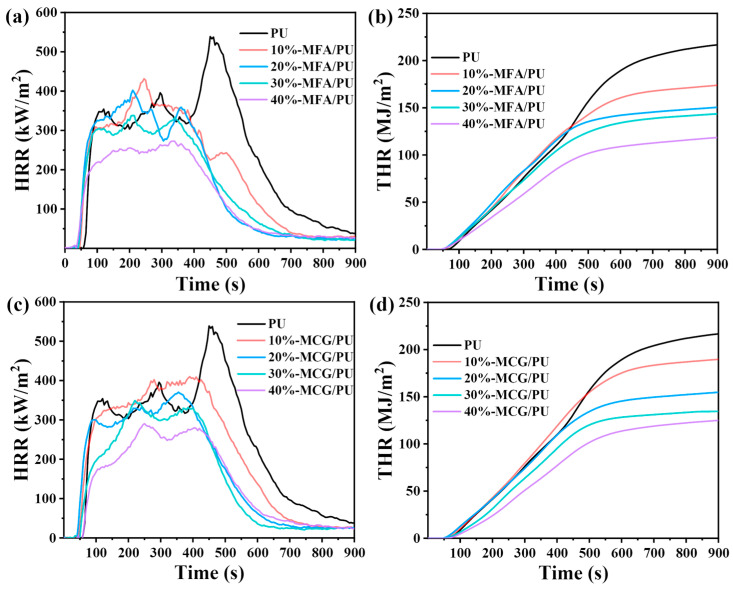
HRR (**a**) and THR (**b**) of MFA/PU composite; HRR (**c**) and THR (**d**) of MCG/PU composite.

The two kinds of composites were subjected to CCT to investigate their combustion behavior further, and the data obtained are listed in Table 5. Important parameters, such as peak heat release rate (PHRR), total heat release (THR), and total smoke production (TSP), can be obtained through CCT [35]. As shown in Figure 7a,c and Table 5, pure PU has three HRR peaks, and its maximum PHRR value is 538.7 kW/m^2^. The samples with MFA and MCG fillers have lower PHRR values than pure PU. The HRR and PHRR peaks of the composites change when the content of the two fillers increases gradually. When the filler content reaches 40%, the HRR peaks of the MFA/PU and MCG/PU composites change into two, and the PHRR values decrease by 49.4% and 46.1% to 272.7 kW/m^2^ and 290.5 kW/m^2^, respectively.

The composites show two or three HRR peaks because of the collapse of the burnt layer, which leads to the release of a large amount of internal combustibles [36]. All samples exhibit multi-peak phenomenon. The first peak can be attributed to the rapid formation of a charred layer on the material’s surface. The charred layer gradually degrades after continuous exposure to high heat, at which time the decrease in HRR is inhibited and the next peak appears during the formation of a new barrier layer [37,38]. At a high filler content, the number of HRR peaks in the two composite materials decreases, indicating that the addition of MFA and MCG is helpful for stabilizing the formation of a coke layer.

Figure 7b,d and Table 5 show that the THR decreases with the increase in the MFA and MCG filler contents. The gradient of the THR curve can be assumed to be representative of fire propagation [39]. Compared with that of PU, the THR curve gradient of the two composites with high filler contents decreases drastically, indicating that the flame propagation speed decelerates mainly because a stable and dense barrier layer structure that limits flame propagation has formed on the sample’s surface. Although the MFA/PU composites have a lower THR than the MCG/PU composites, the gradient of the THR curve from the MCG/PU composites decreases more drastically. This phenomenon shows that both fillers reduce the heat release of materials, and the combustion behavior of MCG may contribute to the formation of a dense barrier structure.

**Figure 8:**
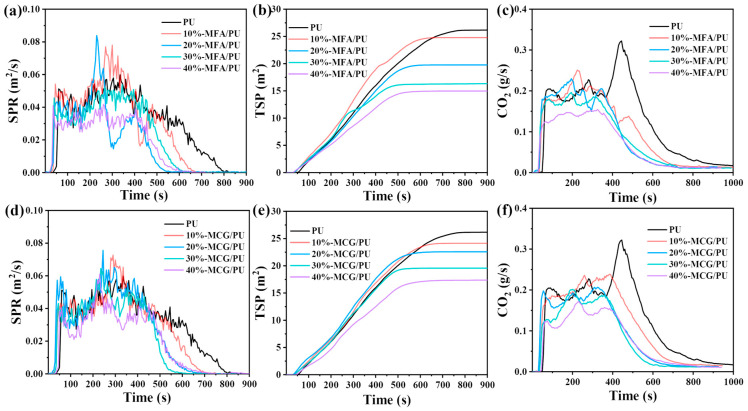
SPR (**a**), TSP (**b**), and CO_2_ generation rate (**c**) of MFA/PU composite; SPR (**d**), TSP (**e**), and CO_2_ production rate (**f**) of MCG/PU composite.

The smoke release rate (SPR), TSP, and CO_2_ production rate of all the samples can be seen in Figure 8. Figure 8a,d show that the smoke release of pure PU lasts for approximately 800 s, and a large amount of smoke is released during combustion. With the increase in the contents of the two fillers, the flue gas release time of the composites decreases, and the SPR curve changes remarkably. When the filler content is less than 30%, the peak values of the SPR from the two composites are higher than that of PU, and the SPR peak of the MCG/PU composites is lower than that of the MFA/PU composites. When the filler content is low, the barrier layer formed by the combustion products and fillers is affected by flue gas release, and the fracture and regeneration of the barrier layer cause the SPR curve of the composites to fluctuate considerably. This phenomenon shows that the filler content affects the stability of the barrier layer and the barrier layer produced by the combustion of the MCG/PU composites is stable. When the composites are burned at a high temperature, the spontaneous combustion behavior of MCG results in the close combination of the combustion products of MCG and the PU matrix. This phenomenon may account for the stability of the barrier layer of the MCG/PU composites during combustion. When the filler content is high, the combination of fillers and combustion products is affected by the filler content, and residual oxides accelerate barrier formation [40]. The rapid formation of a barrier can retard the production of combustible gases and smoke-forming materials in the gas-phase combustion zone [41]. This effect decelerates the combustion of composite materials and reduces the flue gas release rate.

Figure 8b,e and Table 5 show that the addition of two fillers drastically reduces the TSP of the composites. The TSP of pure PU is 26.2 m^2^ and that of the two composites decreases with the increase in the filler content. At the filler content of 40%, the TSP of the MFA/PU and MCG/PU composites is 14.9 m^2^ and 17.4 m^2^, respectively, which is 43.1% and 33.6% lower than that of pure PU, respectively. The smoke suppression effect of MFA and MCG is mainly due to the fact that both fillers contain a variety of oxides, which can quickly form a barrier with combustion products, hinder heat and oxygen exchange, and prevent combustible gas from overflowing [38,42].

The CO_2_ production rates of all the samples are shown in Figure 8c,f. Among the samples, pure PU has the highest CO_2_ release rate. With the increase in the MCG and MFA filler contents, the CO_2_ yield of the two composites decreases gradually during combustion. The CO_2_ production rate of the two composites with the filler mass fraction of 40% is lower than that of pure PU. In addition, after the addition of MFA and MCG, the peak value of the CO_2_ release rate shifts to the left. This change indicates that the addition of MFA and MCG could promote the oxidation of soot particles into CO_2_ and release CO_2_ rapidly. The early release of CO_2_ can dilute the concentration of O_2_ in the combustion zone. This effect is helpful for curbing fire propagation in the early stage and relieving the pressure of fire rescue.

### 3.7. Carbon Residue Analysis

**Figure 9:**
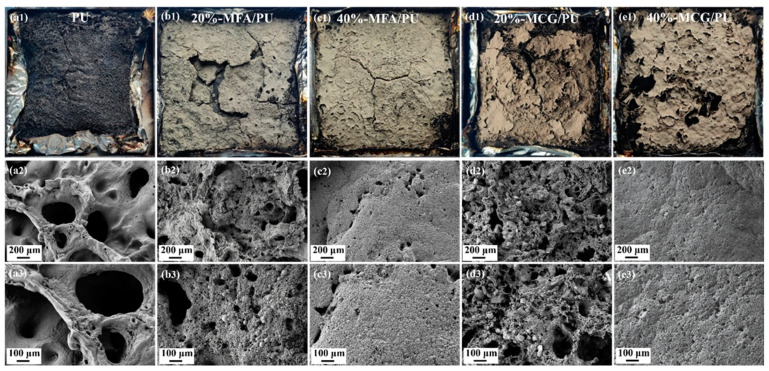
Digital photos and SEM images of the carbon residues of PU (**a**), MFA/PU (**b**,**c**), and MCG/PU (**d**,**e**) composites after CCT.

Figure 9 presents the photographs and SEM morphology images of the residues of the PU, MFA/PU, and MCG/PU composites after CCT. The formation of an effective barrier layer can prevent heat transfer between flames and the combustion matrix and then inhibit the combustion and pyrolysis of the underlying composite [38,43]. Table 5 and Figure 8 show that the residual amount of the two composites increases with the increase in the filler content. Consistent with the SPR curve, the residual layer of the composites with MFA and MCG first breaks and then regenerates, and the morphology of the residues of the MCG/PU composites is more complete. This result proves that the filler content affects the stability of the barrier layer, and the barrier layer formed by the MCG/PU composites is more stable than that formed by the MFA/PU composites.

In addition, the SEM morphology of Figure 9 shows that the carbon layer surface of pure PU is loose and porous. With the increase in the content of two kinds of fillers, the barrier layer morphology of the composites presents a more and more dense structure. This phenomenon shows that the addition of MFA and MCG can promote the formation of a dense barrier layer, and the continuous and dense barrier layer can be used as a barrier to hinder the heat and oxygen exchange during the combustion of composites and inhibit the release of smoke. In addition, when the filler content is 40%, compared with the MFA/PU composites, the barrier layer formed by the MCG/PU composites during combustion is denser. This phenomenon shows that the combustion of MCG is beneficial to the formation of a dense barrier layer.

In general, different from LOI testing, the CCT results indicate that the MFA/PU and MCG/PU composites show a similar flame-retardant behavior in the condensed phase. The combustion of MCG contributes to the formation of a dense barrier layer, which has a positive impact on enhancing the fire safety of the composites.

### 3.8. Flame-Retardant Mechanism

**Figure 10:**
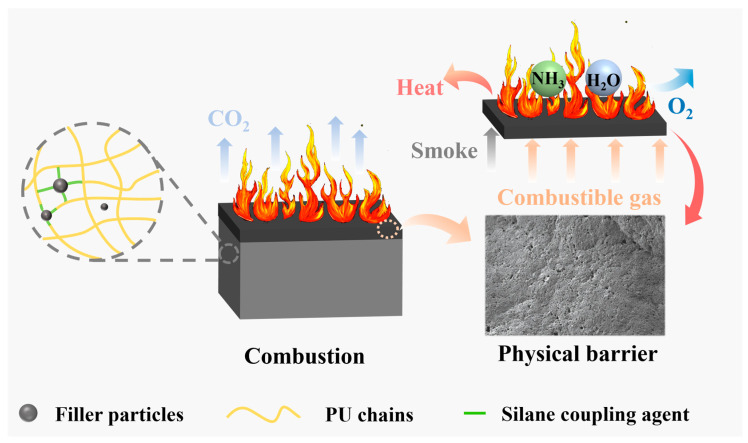
Schematic of the flame-retardant mechanism.

The flame-retardant mechanism of the two composites is presented in Figure 10. Pyrolysis products, such as NH_3_ and water vapor, can consume a considerable amount of heat. At the same time, the addition of MFA and MCG can promote the early release of incombustible CO_2_ and reduce the concentrations of O_2_ and combustible gases. Oxides in MCG and MFA can accelerate the formation of a dense barrier layer. The barrier layer that forms at high temperatures can limit heat and oxygen exchange as well inhibit the release of smoke and combustible gas. In addition, the combustion of MCG is conducive to the formation of a stable and dense barrier layer structure, which is helpful for improving the fire safety of composites.

### 3.9. Water Quality Test

The existing technologies for the utilization of coal-based solid waste have more or less secondary pollution problems. Therefore, the environmental impact of recycling methods for coal-based solid waste must be considered. The 30%-CG/PU and 30%- FA/PU composites were chosen to test their effects on water quality, and the results are shown in Table 6 below.

The results reveal that the leaching concentrations of total hardness, chloride, sulfate, and heavy metal ion content for the 30%-CG/PU and 30%-FA/PU composite materials are lower than the standards for domestic water quality. As previously reported, PU/FA blended under ambient conditions does not release heavy metals at room temperature even under the conditions of long-term high humidity [44]. Therefore, the heavy metal ions in CG and FA would not leak out because they are embedded in PU.

## 4. Conclusions

MFA/PU and MCG/PU composites were prepared by adding MFA and MCG fillers to PU materials. Surface modification can remarkably improve the interfacial compatibility between the fillers and PU matrix. Both kinds of modified coal-based solid waste fillers show the best comprehensive performance at the filler content of 30%. The compressive strengths of MFA/PU and MCG/PU are 84.1 MPa and 46.3 MPa, respectively. The LOIs of the MFA/PU and MCG/PU composites are 29% and 23.5%, respectively, and MFA has advantages in improving the LOIs of the composites. The CCT and SEM results show that the two composites exhibit similar condensed-phase flame-retardant behaviors during combustion, which releases CO_2_ in advance and accelerates the formation of a dense barrier layer. Compared with that of MFA, the combustion of MCG is more conducive to the formation of a stable and dense barrier structure and has a positive effect on the fire safety of reinforced composites. Water quality tests show that heavy metal ions would not leak out from the composites. This characteristic indicates that composites with PU materials are a promising new way for the safe and high-value application of coal-based solid waste.

## Figures and Tables

**Table 1:** Specific formulas of PU, FA/PU, and CG/PU composite materials.

Sample	PPG-200 (g)	RT-305 (g)	PAPI (g)	DBTDL (g)	MFA (g)	MCG (g)
PU	23.75	38.75	37.5	0.15	0	0
10%-MFA/PU	21.37	34.88	33.75	0.15	10	0
20%-MFA/PU	19	31	30	0.15	20	0
30%-MFA/PU	16.63	27.12	26.25	0.15	30	0
40%-MFA/PU	14.25	23.25	22.5	0.15	40	0
10%-MCG/PU	21.37	34.88	33.75	0.15	0	10
20%-MCG/PU	19	31	30	0.15	0	20
30%-MCG/PU	16.63	27.12	26.25	0.15	0	30
40%-MCG/PU	14.25	23.25	22.5	0.15	0	40

**Table 2:** Phase composition of FA and CG was determined by XRD.

Sample	Noncrystalline (%)	Quartz (%)	Kaolin (%)	Illite (%)	Plagioclase (%)	Potassium feldspar (%)	Chlorite (%)	Mullite (%)
CG	22.6	26.9	19.3	12.9	8.4	5.0	4.9	—
FA	69.7	7.5	—	—	—	—	—	22.8

**Table 3:** Compositions of FA and CG was determined by XRF.

Sample	SiO_2_ (%)	Al_2_O_3_ (%)	Fe_2_O_3_ (%)	CaO (%)	K_2_O (%)	SO_3_ (%)	Other (%)	Ignition Loss (%)
FA	54.79	31.43	4.19	3.06	2.42	0.82	3.29	—
CG	54.81	21.95	4.82	1.20	3.21	0.24	4.07	9.7

**Table 4:** Limiting oxygen index of PU, MFA/PU, and MCG/PU composites.

Sample	PU	MFA/PU				MCG/PU			
Filler content (wt%)	—	10	20	30	40	10	20	30	40
LOI (%)	19	22	25	29	28	21	22	23.5	22

**Table 5:** Cone calorimetric test (CCT) data: PHRR, THR, TSP, and residue of PU and analyzed samples of MFA/PU and MCG/PU composites.

Sample	PHRR (kW/m^2^)	THR (kW/m^2^)	TSP (MJ/m^2^)	Residue (%)
PU	538.68	266.6	26.2	10.7
10%-MFA/PU	429.44	182.5	24.8	16.9
20%-MFA/PU	402.02	151.0	19.7	29.2
30%-MFA/PU	337.87	145.6	16.3	37.8
40%-MFA/PU	272.65	119.7	14.9	46.0
10%-MCG/PU	410.03	190.8	24.1	19.3
20%-MCG/PU	370.43	155.4	22.6	26.1
30%-MCG/PU	348.99	134.5	19.6	35.7
40%-MCG/PU	290.50	125.9	17.4	43.9

**Table 6:** Water quality impact test results of 30%-FA/PU and 30%-CG/PU composite materials.

Testing Items	Domestic Water	Water with 30%-FA/PU	Water with 30%-CG/PU	Standard Limit [29]
Total hardness (mg/L)	2.13	2.57	2.18	450
Sulfate ions (mg/L)	62.5	69.2	68.1	250
Chloride (mg/L)	17.5	17.9	18.5	250
Zn (mg/L)	3.35 × 10^−3^	3.84 × 10^−3^	4.77 × 10^−3^	1.0
Pb (mg/L)	1.01 × 10^−5^	1.90 × 10^−5^	1.10 × 10^−5^	0.01
Hg (mg/L)	ND	ND	ND	0.001
As (mg/L)	8.38 × 10^−4^	1.04 × 10^−3^	1.01 × 10^−3^	0.01
Cd (mg/L)	ND	ND	ND	0.005

## Data Availability

The original contributions presented in the study are included in the article/Supplementary Materials; further inquiries can be directed to the corresponding author.

## Supplementary Materials

The following supporting information can be downloaded at: https://www.mdpi.com/article/10.3390/polym16142044/s1, Figure S1: compressive curves of composites filled with different amounts of (a) FA, (b) MFA; Figure S2: compressive curves of composites filled with different amounts of (a) CG, (b) MCG.
