# Supplementary material for: High-Value and Environmentally Friendly Recycling Method for Coal-Based Solid Waste Based on Polyurethane Composite Materials
Source: Polymers (Basel). 2024 Jul 17;16(14):2044. doi: 10.3390/polym16142044 (PMC11281150; doi:10.3390/polym16142044)
Supplement: Supplementary file 1 [file polymers-16-02044-s001.zip › polymers-3084811-supplementary.pdf]

Electronic Supplementary Information

High-Value and Environmentally Friendly Recycling Method for Coal-Based Solid Waste Based on Polyurethane Composite Materials

Xu Li[1],Yang Liu[1], Mingyi Li[1], Sitong Zhang[1], Lan Jia[1,2,*], Fengbo Zhu[1,2] and Wenwen Yu[1]

(1. School of Materials Science and Engineering, Taiyuan University of Technology, Taiyuan 030024, China;

2. Shanxi-Zheda Institute of Advanced Materials and Chemical Engineering, Taiyuan 030000, China)

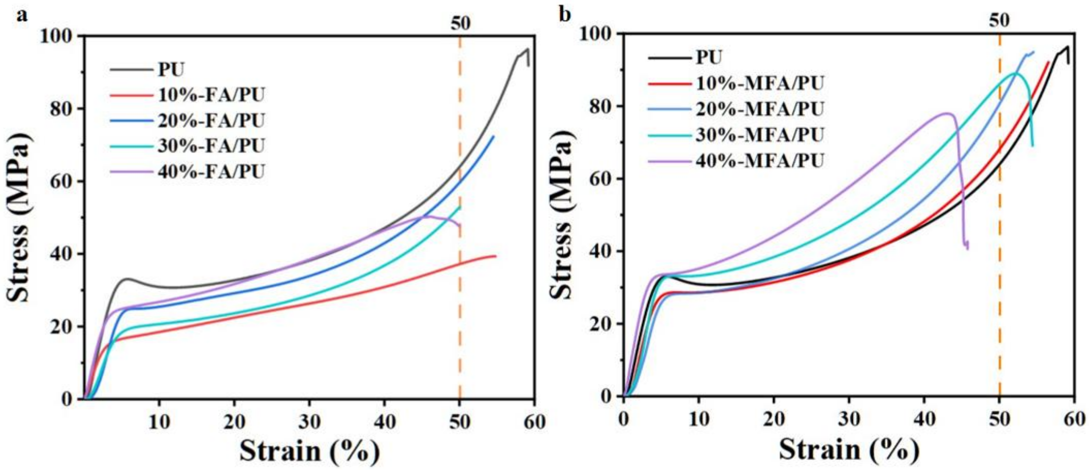

Figure S1. Compressive curves of composites filled with different amounts of (a) FA, (b)MFA

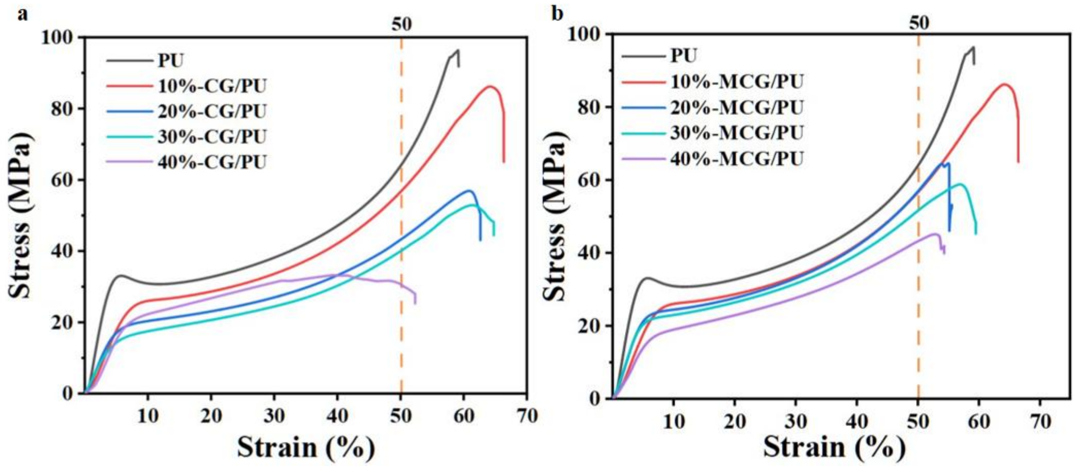

Figure S2. Compressive curves of composites filled with different amounts of (a) CG, (b)MCG
